# Surgical timing and outcomes after volar plate fixation of distal radius fractures: A matched cohort study

**DOI:** 10.1371/journal.pone.0353383

**Published:** 2026-07-15

**Authors:** Christiane Barthel, Michelle Antonia Hinkelmann, Karina Isenmann, Alexander Pieringer, Jonin Serafin Zumsteg, Hans-Christoph Pape, Florin Allemann

**Affiliations:** 1 Department of Traumatology, University Hospital of Zurich, Zurich, Switzerland; 2 Department of Shoulder and Elbow Surgery, Schulthess Klinik, Zurich, Switzerland; 3 University of Zurich, Zurich, Switzerland; Menoufia University, EGYPT

## Abstract

**Background:**

The impact of surgical timing on outcomes in orthopedic trauma surgery remains controversial. While most evidence derives from hip fractures and other major injuries, data on distal radius fractures is limited. This study aimed to assess whether surgical timing influences operative duration, reduction quality, complication rates, and functional outcome after operative treatment of distal radius fractures.

**Methods:**

This single-center, retrospective study included patients aged ≥ 16 years who underwent surgical treatment for distal radius fractures at our hospital between 01.01.2016 and 31.12.2024. Patients without postoperative radiographic follow-up, those treated conservatively, or those managed by other departments were excluded. Patients were grouped according to surgical timing into regular-hours surgery (08:00–16:00) and out-of-hours service. A 1:1 matched cohort analysis was performed with criteria being AO/OTA fracture classification and propensity scoring for age and sex. Analyses were stratified by AO/OTA fracture type (A, B, and C). Outcomes included operative time, radiographic reduction quality, complication rates, and functional outcome assessed by postoperative range of motion.

**Results:**

A total of 660 patients were included, of whom 434 underwent surgery during regular hospital hours and 226 during out-of-hours service. From the out-of-hours cohort, matching was performed using an exact match for fracture morphology according to the AO/OTA classification and propensity score matching for age and sex, resulting in two well-balanced 1:1 matched groups comprising 128 patients each. Across both groups, operative duration, reduction quality, complication rates, and functional outcome assessed by postoperative range of motion did not differ between regular-hours and out-of-hours surgery.

**Conclusion:**

Surgical timing was not associated with differences in operative duration, reduction quality, complication rates, or functional outcome after operative treatment of distal radius fractures. Within this matched cohort, no measurable differences were detected between regular-hours and out-of-hours surgery. As the study was not designed to establish equivalence, these findings indicate the absence of a detectable difference in this specific institutional setting and should not be interpreted as support for routine out-of-hours fixation.

## Background

Distal radius fractures are among the most common fractures requiring surgical treatment in adults and represent a distinct clinical entity with specific surgical techniques, patient characteristics, and outcome measures [1,2]. Surgical timing, commonly evaluated using time-to-surgery metrics, has shown inconsistent associations with clinical and radiological outcomes [3,4]. The influence of surgical timing on outcomes in orthopedic trauma surgery has been widely investigated and most authors recommend against operating “everything” at night [5,6].

However, most studies comparing procedures performed during regular daytime hours with those conducted during out-of-hours or on-call service report no major differences in clinical outcomes [7,8].

Previous research has primarily focused on hip fractures, spine surgery, and pediatric trauma, while evidence for other common fracture types is limited [9–11]. Outcomes potentially influenced by surgical timing include operative time, technical quality of fracture reduction, complication rates, and functional recovery. These parameters are of particular relevance in orthopedic trauma surgery, where procedures are frequently performed outside regular working hours [10]. Distal radius fractures represent one of the most frequently operatively treated fractures in adult trauma care [12,13] and encompass a broad spectrum of injury complexity, including technically demanding intra-articular fracture patterns. Their frequent management during both regular working hours and out-of-hours service makes them a clinically relevant model for evaluating the potential influence of surgical timing in routine trauma practice.

Therefore, the aim of this study was to evaluate whether surgical timing affects operative and clinical outcomes after surgical treatment of distal radius fractures. We examined whether surgical timing – regular hospital hours versus out-of-hours service – affects operative duration, radiographic reduction quality, complication rates, and functional outcome assessed by range of motion in patients undergoing surgical treatment for distal radius fractures.

## Materials and methods

### Patients and data

This single-center, retrospective study was conducted at our institution and included patients treated for distal radius fractures between January 1, 2016, and December 31, 2024. Data were accessed for research purposes between February 15, 2025, and March 15, 2025.

Inclusion criteria were surgical treatment of a distal radius fracture (excluding cases requiring emergency intervention due to neurovascular compromise or plastic surgical involvement), age ≥ 16 years at the time of surgery, provision of informed consent, and availability of complete follow-up data.

Exclusion criteria included absence of postoperative follow-up or radiographs, conservative treatment, and patients managed by other departments. Only patients with sufficient follow-up data were included in the matched cohort analysis. Data on fracture morphology, surgical procedures, complications, follow-up visits, and radiological imaging were extracted from the institutional clinical database.

Functional outcome was assessed based on wrist range of motion (ROM), including extension and flexion in degrees, measured at the last available follow-up visit. Measurements were obtained during routine clinical examinations by an upper extremity surgeon. The reported values represent absolute ROM in degrees, with higher values indicating better functional outcome.

### Matching

Patients were allocated to one of two groups according to the timing of surgery: regular hospital hours (08:00–16:00) and out-of-hours service, defined as procedures performed between 16:00 and 08:00.

A matched cohort design was applied, pairing patients from the out-of-hours group with those from the regular-hours group. Matching was performed using exact matching for AO/OTA fracture classification and propensity score matching for age and sex (nearest-neighbor matching without replacement). Only patients with successful matches were included in the final analysis.

Following matching, patients were stratified by AO/OTA fracture type (A, B, and C), and all analyses were repeated within each subgroup to assess whether surgical timing had a differential impact across levels of fracture complexity. To further explore potential differences within the out-of-hours cohort, an exploratory subgroup analysis comparing evening procedures (16:00–22:00) and overnight procedures (22:00–08:00) was performed.

### Definitions

Reduction outcome was assessed using five radiographic parameters measured on conventional biplanar radiographs. Measurements were performed by two senior upper extremity surgeons and three orthopedic residents. Assessors were not formally blinded to surgical timing; however, surgical timing was not actively considered during evaluation.

In the absence of a widely accepted scoring system for distal radius fractures, a point-based composite score was applied to compare reduction quality. One point was awarded for each parameter if the postoperative measurement fell within predefined acceptable ranges based on established instability criteria and outcome predictors (Table 1).

**Table 1:** Criterion for Point Assignment.

Parameter	Radiograph	Cutoff for 1 point
Volar Tilt	Lateral	≥ 0°
Radial Inclination	PA	≥ 20°
Radial Shortening	PA	≤ 3 mm
Ulnar Variance	PA	≤ + 3 mm
Joint Step-off	PA/Lateral	≤ 1 mm

Volar tilt was considered satisfactory at ≥0°, radial inclination at ≥20°, radial shortening at ≤3 mm (corresponding to a minimum radial height of ≥9 mm), ulnar variance at ≤+3 mm, and articular congruity when the step-off was ≤ 1 mm. A maximum of five points could be achieved, with higher values indicating superior reduction quality.

### Statistics

Statistical analyses were performed using Python™. Descriptive statistics are presented as means with standard deviations (SD) or medians with interquartile ranges (IQR) for continuous variables, and as frequencies with percentages for categorical variables.

Group comparisons were conducted using the Mann–Whitney U test for continuous variables and the Chi-square test for categorical variables. Propensity scores were estimated using logistic regression based on age and sex, while exact matching was applied for AO/OTA fracture classification.

Although a matched design was applied, statistical analyses were performed at the group level using unpaired tests, as the matching procedure was intended to achieve comparability between cohorts rather than to assess within-pair differences.

All statistical tests were two-tailed, and statistical significance was defined as p < 0.05.

### Ethics approval and consent to participate

The study was conducted in accordance with the Declaration of Helsinki and approved by the local ethics committee of the Canton Zürich (Study-Nr 2024−01809). All patients had provided written informed consent for the use of their medical data for research purposes through the institution’s general consent form.

The study was retrospective in nature, and all data were pseudonymized, with the re-identification key stored separately in accordance with institutional data protection policies. No minors were included; therefore, no additional study-specific consent was required.

## Results

**Fig 1:**
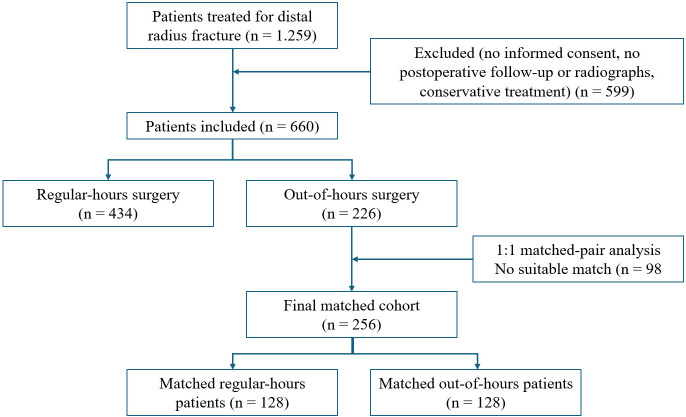
Flow diagram illustrating patient selection, exclusion, 1:1 matched cohort analysis, and final study cohort.

Between January 1, 2016, and December 31, 2024, a total of 1,259 patients were treated for distal radius fractures at our institution. Of these, 660 patients met the inclusion criteria and were included in the analysis. Among these, 434 patients (65.8%) underwent surgery during regular hospital hours (08:00–16:00), while 226 patients (34.2%) were treated during out-of-hours service. (Fig 1)

To ensure comparability between groups, a matched cohort analysis was performed using exact matching for AO/OTA fracture classification and propensity score matching for age and sex. This approach yielded 128 well-balanced 1:1 matched pairs, with 128 patients per group. Baseline characteristics were well balanced between groups (Table 2). Median follow-up was 12 months in both groups. Temporary external fixation was more frequently used in the regular-hours group (12.5% vs. 4.7%, p = 0.043).

**Table 2:** Baseline characteristics of the matched cohort.

Characteristic	Regular hours	Out-of-hours service
**Matched pairs (n)**	128	128
**Age (years)**	52.0 ± 19.3	52.0 ± 19.3
**Sex, n**		
Female	73	72
Male	55	56
**AO/OTA, n**		
Type A	46	46
Type B	12	12
Type C	70	70
**Laterality, n**		
Left	61	59
Right	67	69
**Initial Fixateur externe treatment**	16 (12.5%)	6 (4.7%)

In the matched cohort, operative time did not differ significantly between surgeries performed during regular hospital hours and those performed during out-of-hours service. In AO type A fractures (46 matched pairs), median operative time was 75 minutes during out-of-hours service and 66.5 minutes during regular hours, with no statistically significant difference (p = 0.29). Similarly, no significant differences in operative duration were observed for AO type B fractures (85.5 minutes during regular hours vs. 70.5 minutes out-of-hours, p = 0.38) or AO type C fractures (78.0 minutes during regular hours vs. 77.0 minutes out-of-hours, p = 0.60) (Table 3).

**Table 3:** Operative time.

AO/OTA Group	n (pairs)	Regular hours, min (median, IQR)	Out-of-hours service, min (median, IQR)	p-value
A	46	66.5 (58.3–87.3)	75.0 (55.3–90.8)	0.290
B	12	85.5 (68.0–110.3)	70.5 (60.0–98.3)	0.380
C	70	78.0 (67.3–100.0)	77.0 (65.5–95.5)	0.600

To further assess whether the timing within the out-of-hours cohort influenced outcomes, an exploratory subgroup analysis comparing evening procedures (16:00–22:00; n = 57) and overnight procedures (22:00–08:00; n = 71) was performed. No statistically significant differences were observed between these subgroups regarding operative time (72 vs. 79 minutes, p = 0.085), reduction quality (mean reduction score 4.62 vs. 4.65, p=1.000), complication rates (6.3% vs. 3.9%, p=0.550), or postoperative range of motion (S1 Table).

Reduction outcomes were comparable between surgical timing groups across all AO/OTA fracture types, with predefined radiological criteria met at high and similar rates in both groups (Table 4). In AO type A and B fractures, reduction scores were high in both groups, with median values of 5 and no significant differences between groups. In AO type C fractures, reduction scores remained similarly high, with mean scores of 4.71 during regular hours and 4.68 during out-of-hours service (median 5 in both groups; p = 1.00). No statistically significant differences were observed in any individual reduction parameter.

**Table 4:** Overall reduction score (0–5) in the matched cohort.

Outcome	n (pairs)	Regular hours (mean ± SD, median)	Out-of-hours service (mean ± SD, median)	p-value
Overall	128	4.62 ± 0.69 (5)	4.65 ± 0.65 (5)	1.00
AO/OTA Type A	46	4.80 ± 0.45 (5)	4.72 ± 0.54 (5)	1.00
AO/OTA Type B	12	4.50 ± 0.80 (5)	4.50 ± 0.90 (5)	1.00
AO/OTA Type C	70	4.51 ± 0.78 (5)	4.63 ± 0.66 (5)	1.00

The complications observed included infections, CRPS, tendon ruptures, compartment syndrome, nerve lesions, and implant malposition. These complication rates were low and comparable between groups across all AO/OTA fracture types. In AO type A fractures, complications occurred in two cases in each group (p = 1.00), while in AO type B fractures, one complication was observed per group (p = 1.00). In AO type C fractures, complications occurred in five cases during regular hours and two cases during out-of-hours service, without a statistically significant difference (p ≈ 0.45). (Table 5)

**Table 5:** Complications in the matched cohort.

Outcome	n (pairs)	Regular hours n (%)	Out-of-hours service n (%)	p-value
Overall	128	8 (6.3%)	5 (3.9%)	0.550
AO/OTA Type A	46	2 (4.3%)	2 (4.3%)	1.000
AO/OTA Type B	12	1 (8.3%)	1 (8.3%)	1.000
AO/OTA Type C	70	5 (7.1%)	2 (2.9%)	0.450

Functional outcome, assessed as wrist range of motion at final follow-up, did not differ significantly between surgeries performed during regular hospital hours and those performed during out-of-hours service (Table 6). Overall, mean wrist extension and flexion were comparable between groups (62.4° vs. 60.0°, p = 0.403; and 64.2° vs. 62.4°, p = 0.726, respectively). No statistically significant differences were observed across AO/OTA fracture types, although numerically lower values were noted in AO type B fractures treated out-of-hours.

**Table 6:** Range of motion at last follow-up.

Outcome	n (pairs)	Regular hours (mean)	Out-of-hours service (mean)	p-value
**Extension (°)**	128	62.4	60	0.403
**Flexion (°)**	128	64.2	62.4	0.726
**AO/OTA Type A**	46			
Extension (°)		66.5	62.2	0.343
Flexion (°)		64.2	63.9	0.913
**AO/OTA Type B**	12			
Extension (°)		67.3	57.5	0.333
Flexion (°)		69.1	57.5	0.507
**AO/OTA Type C**	70			
Extension (°)		59.1	59	0.972
Flexion (°)		63.3	62.4	0.799

## Discussion

Distal radius fractures are among the most common adult fractures with an incidence of 207.7 per 100,000 person-years, showing a bimodal distribution with peaks in children and elderly women [12,13]. Extraarticular fractures predominate (65–69%), and 15–26% undergo operative treatment, primarily with volar plating [14,15]. The socioeconomic burden is substantial, though comprehensive cost data remain limited. While neurovascular emergencies mandate urgent treatment [16–18], the optimal timing for stable fractures remains controversial. In this context, the present findings demonstrate comparable outcomes between regular-hours and out-of-hours surgery across all AO fracture types.

### Operative duration

In the present matched cohort analysis, operative duration was not influenced by surgical timing across all AO fracture types, supporting the concept that perioperative efficiency can be maintained during out-of-hours surgery even in more complex fracture patterns. This finding is in line with adult trauma literature reporting comparable operative times between regular-hours and out-of-hours procedures. Santangelo et al. (2023) examined 241 urgent spine procedures and observed no significant difference in operative duration for urgent spine surgery performed during daytime versus out-of-hours [11]. Similar results were reported by Yeo et al. (2021), who analyzed 903 hip fracture operations and found no significant difference in surgical duration between office hours (n = 693) and out-of-hours (n = 210) [19]. Saygılı et al. (2022) reported equivalent operative times for intramedullary nailing of tibial shaft fractures in 57 patients (p = 0.419) [20]. Reports of prolonged operative times or inferior outcomes out-of-hours predominantly stem from pediatric trauma settings and have been attributed to reduced senior supervision rather than timing itself [8]. Together, these findings suggest that institutional staffing structures and experience, rather than the time of surgery, are the primary determinants of perioperative efficiency [8].

### Reduction quality

The threshold values for our five-parameter scoring system were selected based on established anatomical norms and clinical evidence. A threshold of ≥0° volar tilt was selected to ensure restoration of neutral or volar alignment. Reported acceptable ranges for palmar tilt lie between approximately 1° and 21°, based on OTA-derived reference values reported in the literature [21]. Physiological values are generally described within a positive range, supporting the restoration of a non-dorsal alignment [22]. Radial inclination ≥20° was selected based on clinical evidence, as Symonette et al. demonstrated a significantly increased risk of poor outcome when radial inclination falls below 20° [23]. Radial shortening ≤ 3 mm was selected based on AAOS guidelines emphasizing strict limits for optimal outcomes [24,25]. This threshold corresponds to restoration of radial height within an acceptable range of approximately 9–12 mm, as reported by Rajadurai et al. [26], given that physiological radial height is typically around 12 mm in the general population [27,28]. Ulnar variance ≤+3 mm was derived from Labbe et al. (2024) [29], who demonstrated that >3 mm variance significantly compromised outcomes (RR 0.558, p < 0.0001). Finally, articular step-off ≤1 mm aligns with AAOS criteria [25] and the classic work by Knirk and Jupiter (1986) showing increased arthritis risk with >1–2 mm displacement. This point-based composite score (0–5 points) enhances clinical applicability and reduces measurement variability.

Reduction quality was comparable across timing groups for all AO types, with maximum scores in type A/B fractures and near-identical scores in type C fractures. This aligns with evidence demonstrating that fracture complexity—not surgical timing—primarily determines reduction difficulty [5,6]. Karagoz et al. (2022) reported no difference in reduction quality for 124 femoral neck fractures between daytime and out-of-hours groups [7]. The pediatric literature shows inferior reduction quality out-of-hours when supervision is inadequate [4], underscoring that institutional structure and expertise—rather than time of day—determine outcomes.

### Complication rates

Complication rates appear largely independent of surgical timing, in line with adult trauma literature. Santangelo et al. (2023) found no significant differences in 30-day return to OR (14.3% vs 6.8%, p = 0.09), readmission (2.0% vs 6.3%, p = 0.24), or mortality (4.1% vs 7.3%, p = 0.42) in 241 spine procedures [11]. Yeo et al. (2021) reported equivalent complication rates in 903 hip fracture patients [19].

In contrast, Halvachizadeh et al. (2019) analyzed 31,692 orthopedic trauma patients from a Swiss national database and reported increased general complication rates for afternoon (OR 1.22, p = 0.006) and nighttime (OR 1.51, p = 0.021) surgery, which the authors attributed to surgeon fatigue, differences in case severity, and institutional staffing patterns rather than timing itself. These findings may not be directly transferable to isolated distal radius fractures, as nighttime cohorts in large trauma registries often include more severely injured patients. In the present study, temporary external fixation was significantly more frequently used in the regular-hours group, suggesting that more complex fractures and potentially more severely injured patients may have been managed with a staged approach. In some cases, this may reflect a damage control strategy, with initial temporary stabilization performed out-of-hours and definitive fixation deferred to daytime surgery. However, despite this potential selection bias, no differences in complication rates or other outcomes were observed between groups.

Within the distal radius literature, complication rates after volar plate fixation are more strongly associated with patient-related factors and intraoperative technical parameters than with surgical timing [7,30], further supporting our findings. The pediatric literature likewise reports inferior reduction quality after-hours when supervision is inadequate [8], underscoring that institutional structure and surgical expertise—rather than time of day—are key determinants of outcomes. Accordingly, the present findings should be interpreted within the context of a structured shift-duty system and do not represent a direct assessment of surgeon fatigue. Generalizability to institutions with different staffing models may therefore be limited. Notably, an exploratory subgroup analysis within the out-of-hours cohort did not reveal significant differences between evening and overnight procedures, suggesting that the observed findings were consistent across different out-of-hours periods.

### Range of motion outcomes

Functional recovery after distal radius fracture fixation is primarily influenced by fracture stability, fixation quality, and postoperative rehabilitation, as demonstrated in previous studies [24,31,32]. The role of surgical timing appears to be of lesser importance. Prior studies demonstrate comparable range of motion once stable internal fixation and standardized rehabilitation protocols are achieved, irrespective of fixation strategy or surgical timing [24,31–33]. Similar observations have been reported in other adult trauma populations, including hip fracture and spine surgery, whereas inferior functional outcomes after out-of-hours service are mainly described in pediatric settings and are attributed to reduction failure rather than timing itself [8,11,20].

### Limitations

This study has several limitations.

First, although exact matching for AO/OTA fracture classification and propensity score matching for age and sex were applied, residual confounding due to unmeasured variables—such as surgeon experience, case urgency, patient comorbidities, or ASA classification—cannot be fully excluded.

Second, treatment pathways for more complex fracture patterns may have influenced group allocation. In selected cases, temporary stabilization (e.g., external fixation) may have been performed during out-of-hours service, with definitive fixation deferred to regular hours. This staged approach may reflect a damage control strategy in more severely injured patients and could lead to an underrepresentation of complex cases in the out-of-hours definitive fixation group. In line with this, staged treatment was more frequently observed in the regular-hours cohort, suggesting a potential selection of more complex cases into this group.

Third, no formal a priori power calculation was performed. Given the low event rates and limited subgroup sizes, particularly in AO type B fractures, the study may be underpowered to detect small but clinically meaningful differences. In addition, the subgroup analysis comparing evening and overnight out-of-hours procedures was exploratory in nature and not specifically powered as a primary comparison. Accordingly, the study was not designed as an equivalence or non-inferiority study, and the absence of statistically significant differences should not be interpreted as evidence of equivalence between groups. No minimal clinically important differences were prespecified for the investigated outcomes; therefore, the clinical relevance of small observed between-group differences cannot be definitively established. Furthermore, out-of-hours procedures were classified solely according to the time of surgery; weekends and public holidays were not recorded as separate categories. Consequently, a more detailed subdivision into weekday, weekend, and public-holiday procedures was not possible.

Fourth, radiographic reduction quality was assessed using conventional radiographs, which may not detect subtle differences in articular congruity or alignment identifiable on advanced imaging. In addition, interobserver variability cannot be fully excluded, and assessors were not formally blinded to surgical timing, which may introduce observer bias.

Fifth, functional outcome was assessed based on clinical examination by an upper extremity surgeon. While range of motion is an objective and clinically relevant parameter, it may not fully capture patient-reported outcomes or quality of life. In addition, the selected endpoints may not be sufficiently sensitive to detect subtle but clinically relevant differences, and more comprehensive measures such as patient-reported outcomes, grip strength, pain, fluoroscopy time, implant-positioning accuracy, reoperation rates, and return-to-work outcomes were not systematically available in this retrospective cohort.

Sixth, this was a single-center study conducted at a tertiary trauma center with established workflows and experienced surgical teams, which may limit generalizability to institutions with different staffing models. Notably, the study setting employs a dedicated night-duty system, meaning surgeons performing out-of-hours procedures typically start their shifts in the evening rather than operating after a full daytime workload. This organizational structure may mitigate fatigue-related effects. Consequently, this study evaluates the effect of surgical timing rather than directly assessing surgeon fatigue, and the findings should not be extrapolated to institutions in which out-of-hours procedures are performed by surgeons who have already completed a full daytime workload.

Finally, follow-up duration was limited to available clinical documentation and may not capture late complications or long-term functional differences.

## Conclusion

In this matched cohort analysis, no statistically significant differences were observed between regular-hours and out-of-hours surgery with regard to operative duration, reduction quality, intra- and postoperative complication rates, or functional outcome assessed by an upper extremity surgeon at defined follow-up time points.

Within this matched cohort, no measurable differences were detected between regular-hours and out-of-hours surgery. As the study was not designed to establish equivalence, these findings indicate the absence of a detectable difference in this specific institutional setting. Rather, they suggest that selected cases may be managed flexibly within a comparable organizational framework without measurable differences in the investigated outcomes and should not be interpreted as support for routine out-of-hours or nighttime surgery. The findings should be interpreted within the context of distal radius fracture surgery performed in a structured shift-duty system and should not be extrapolated to more complex trauma procedures or different staffing models.

## Supporting information

S1 Table Exploratory comparison of evening (16:00–22:00) versus overnight (22:00–08:00) out-of-hours surgery in the matched cohort. (DOCX)

## Abbreviation:

AO/OTA: Arbeitsgemeinschaft für Osteosynthesefragen/ Orthopaedic Trauma Association
DRF: distal radius fracture
ROM: range of motion
CRPS: complex regional pain syndrome
