# Supplementary material for: Surgical timing and outcomes after volar plate fixation of distal radius fractures: A matched cohort study
Source: PLoS One. 2026 Jul 15;21(7):e0353383. doi: 10.1371/journal.pone.0353383 (PMC13372144; doi:10.1371/journal.pone.0353383)
Supplement: S1 Table — (DOCX) [file pone.0353383.s001.docx]

**Supplementary**

S1 Table: Exploratory comparison of evening (16:00–22:00) versus overnight (22:00–08:00) out-of-hours surgery in the matched cohort

|  | **Evening 16:00–22:00 (n=57)** | **Night 22:00–08:00 (n=71)** | **p-value** |
| --- | --- | --- | --- |
| Operative time, min (median, IQR) | 72 (59–91) | 79 (67–98) | 0.085 |
| Reduction score (mean ± SD, median) | 4.58 ± 0.71, 5 | 4.70 ± 0.60, 5 | 0.300 |
| Complications, n (%) | 2 (3.5%) | 3 (4.2%) | 1.000 |
| Extension at follow-up, mean ± SD (°) | 60.3 ± 19.3° | 59.9 ± 20.3° | 0.942 |
| Flexion at follow-up, mean ± SD (°) | 62.4 ± 19.3° | 62.5 ± 20.0° | 0.847 |
